# Charlotte Brontë—A Psychological Study

**Published:** 1858-04-01

**Authors:** 


### Art. IV.—CHARLOTTE BRONTË—A PSYCHOLOGICAL STUDY.

> "Kaum herrscht noch ein Zweifel darüber, dass der Geist nicht so, wie wir ihn im gebildeten Menschen der Betrachtung unterwerfen Können, als etwas Fertiges Ausgebildetes bei der Geburt des Menschen mit in die Erscheinung tritt; dass vielmehr nur die Möglichkeit der Entwickelung bis zur höchsten Vollkommenheit dem Menschen innewohnt; dass der gebildete Menschengeist somit ein Product sei der Erziehung, wie solche bewirkt wird durch den innewohnenden eigenen Bildungstrieb, durch die umgebende Natur, durch die gebildete Vernunft, d. h. durch díe absichtliche, von schon gebildeten Menschen ausgehende Erziehung, wie endlich durch die Schicksale und den Lenker und Leiter derselben."

THE die does not create the metal, but it makes the current coin, and defines the value that must be attached to it in the world, and the influence that it will exert upon it. So circumstances do not *make* character, but mould it and modify its developments; giving it, not its more or less vigorous vitality, but determining in great measure the direction in which this force shall be manifested. The ivy will find its way to the light through the stone wall, but its course will be longer or shorter, according to the tortuosities which it has to encounter in its passage.

The mode in which the concrete mind is developed and manifested to the world is influenced, or rather determined, by antecedents, which may be summed up under one head,—that of Hereditary Influence: and surrounding circumstances; which include the physical medium into which it is forced—*i.e.*, the nature of the climate, the soil, and the scenery; and the moral medium, including the period, the social, political, and intellectual condition of the community of which it is to be a member, the class of developed mind with which it comes in habitual or casual contact passively; and the active influence exerted by such minds upon it in the process of education and instruction which it undergoes. To these must be added, as not the least operative, that combination of physical and moral agencies which depends upon the state of poverty or wealth amid which it flourishes or languishes. All these are of potent energy; for there is probably no event occurring between the moment of conception and that of the final dissolution of the body, which is without some defined dynamic influence upon mental development, whether such event be of a purely corporeal or a spiritual nature. What is the primary condition of mind, we have no means of ascertaining. Is mind in its essence strictly the same, and dependent for the varieties of its manifestation entirely upon organization and external influences? Are there original differences in mental faculties and capacities previous to any influences from within or without? Such questions we cannot decide. There are those who would answer the first question in the affirmative; who consider that the immaterial essence is the same in all men; that there is originally an inherent capacity in all for the development of the highest grades of intellectual eminence; that this capacity is the birthright and possession of every viable child; and that the differences observed between the widely-separated orders of intelligence depend upon causes partly affecting the organization, such as hereditary influences, food, climate, certain diseases, &c., and partly directly operative upon the intellect, as through varied moral and intellectual training.

From the very nature of the question, no solution is possible, save through the subtleties of metaphysical speculation; for we know nothing of spiritual essences except through their manifestations; and these manifestations are solely through the medium of organization. Practically, then, our investigations must necessarily be limited to the observation of the conditions and capabilities of the organism in relation to mental development.

By far the most powerful and remarkable of the formative influences in the production of character and individuality is hereditary tendency. Not more striking is the feature-likeness between parent and child, than is the resemblance, in a great many instances, between their respective psychical manifestations. We see genius or stupidity propagated generation after generation, as we see the colour of the hair or the form of any particular feature. And not only these general characteristics, but we observe an inheritance of particular dispositions and tendencies, and even, in not a few instances, of acquired habits. This latter is particularly illustrated in some animals, when the results of artificial culture have become in some degree hereditary. The descendants of dogs trained to particular purposes are much more easily educated than others of similar breed, that have not had trained parents. It is most important to observe, also, that vice and tendency to particular forms of crime are hereditary also; and this not only when the vice has been (so to speak) congenital, but when it has been acquired and become habitual. Thus, the confirmed drunkard is likely to have drunken children; and every form of sensuality is liable to be reproduced in the succeeding generation. But even in this very brief and imperfect sketch of the influence of ancestry, we must not omit to mention the development of remarkable ability which is sometimes observed, not as a direct inheritance, but as one of the concomitants of the expiring energies of a degenerate or degenerating race—a phenomenon to which we lately alluded as the "phosphorescence of decay."

The physical medium is, without doubt, of great importance in the formation of character, although it is only when we observe its influence in what may be called extreme cases that we can fully recognise its force. In the districts where cretinism and idiocy are endemic, low forms of mental development are propagated from generation to generation, until the reproductive force is lost; yet if one of these children—one link of this melancholy chain—be taken from birth, and removed to a healthy district, the entail of constitutional vice will be broken; and not only the physical condition will ascend one degree or more towards its normal state, but the faculties of the mind will be improved to a point far above that of its ancestry. We cannot doubt that, within the limits of healthy formation, the same influences are operative in the production of modified organisms, and distinctly sealed minds, though in a degree less marked and obvious; for not more contrasted are the characters of the sleepy, imaginative Asiatic, and the hardy, practical, adventurous inhabitant of our northern latitudes, than are those of our wild moorland districts as compared with those of smoky and crowded cities.

The moral medium is, perhaps, of still greater direct importance in the formation of mind than the physical; and its effects are so obvious as to require but little illustration. The early tone of thought, the fundamental ideas, the habitual bent of the mind, will necessarily be derived from early associations, and will have the stamp and impress of the society in which the first years are passed. And these will never be lost; however after circumstances may be changed, these, the first and most potent impressions will remain, consciously or unconsciously, to tinge the whole future life and character. For instance, the superstition which may mingle with the earliest teachings of a child, may be neutralized by reason in after-days, but the tendency will never be lost.

The period of a country's history and development, and the political aspects of the times, exert a most powerful determining influence upon individual character. This branch of the subject is much too extensive to treat casually; we shall, therefore, merely enumerate it amongst the most potent of causes, referring for illustration to all the great critical times, in our own country especially, where the crises of history are attended by the appearance of names that of themselves would make or mark epochs. The Elizabethan age, when England was passing (so to speak) from ancient to modern civilization, will furnish a good example of our meaning.

The influence of education, as ordinarily conducted, is more exerted upon the contents of the mind than upon its formation; yet, according as it is good or bad, the good or evil qualities of the mind will be drawn out and put in exercise; and it will in some measure determine how far the individual shall be associated with his fellow-creatures by the bonds of good-will and sympathy, or become reserved, repellent, isolated, and suspicious. Even this, however, is limited in its operation; for, to an acutely perceptive mind, the defects of a system may be so apparent as to produce a directly opposite result to that which might be expected from the data; and the character may, by means of great internal energy, be formed rather upon what is wanting in the course of education, than what is present—may, in fact, become the complement or supplement of the system.

But little that is certain and definite can be predicated of the influence which poverty exerts upon mental development. It seems to depend chiefly upon the type of mind, whether it acts as a stimulant, or an utter bar to mental exertion and improvement.

There are, however, two secondary results which must necessarily accrue—it must, to a great extent, limit and control the *practical* expansion of sympathy, and the benevolent emotions generally; and it must at the same time limit the experience and knowledge of the varied forms and conditions of life, by preventing any extended intercourse with the world at large, either through travel or free access to all societies. Hence the knowledge of character will be founded upon contracted data; and, however deep and acute may be the insight, the *range* will be comparatively small.

Such are the circumstances which influence and mould the private life of all, and the public character of the great majority. It is most especially in relation to authorship that we are at present interested in tracing the precise results. Some few there are of a genius so exalted that its emanations exhibit few or no traces of its antecedents. In the plays of our immortal dramatist, we search in vain for any trace of the strolling actor, the deer-stealer, the vagabond, the husband of Ann Hathaway, the man of property, or the country gentleman and magistrate. In the gorgeous scenery of the "Paradise Lost," we have no indication of the bard; there is no blindness, no fierce contest of party wrath, no ingratitude, no home misery; there is none of the fiery sectary—none of the anarchy of the age. There are others of temperament so indifferent as almost to separate the intellect from the outer life; yet when we hear Goethe's unmoved "Oh!" on being told that the Allies had entered Paris, and see Sir Thomas Brown quietly writing his treatise on popular fallacies in the midst of the crash of a disorganizing state, we feel that this is abnormal—that it is not humanity. In Dante, in Petrarch, in Ariosto,—in Voltaire, Byron, Shelley,—in a crowd of others whose names rank amongst the greatest and noblest of writers, whatever may be the theme, we can trace the man. In the "Purgatorio," we find constant allusions to the political errors of the day; in the "Paradiso," the same; so in the others we hear the wailings of the wounded spirit, the ravings of madness, the soft accents of love, the boiling wrath of political or social disappointment, the covert scorn of infidelity, the sneer of universal scepticism, the aspirations after utopian forms of society; and we know then that the man has spoken as well as the poet.

In general, these glimpses of personality and individuality are at most but thinly scattered, or form but a small proportion of the entire works of the author. It is rare to meet with the whole mind in the work; still more rare to be able to trace the entire development of the mind to natural causes—*i.e.*, to antecedents and circumstances which would naturally be expected to produce such a result in ordinary course. Such is, however, the case in

Charlotte Brontë, the formation of whose mind and character we propose to investigate in the following pages. Her most characteristic work, "Jane Eyre," contains a psychical transcript of herself, though the canvas is stretched on a framework of different form to the original; and each point and trait of this character can easily, and without straining, be seen to be due to influences identical with, or analogous to, those to which we have briefly alluded above.

The outlines of her uneventful life are well known through Mrs. Gaskell's graphic pen: the early, almost complete orphanage—the weary, unsocial childhood—the still more weary youth—the hopeless years of fruitless waiting and longing—the patient, trustful perseverance, and almost dogged energy—the brief period of fame, with its few delights, and its many cares and bitternesses—the wearing, wasting toil—the few short months of happiness—the early death—all combine to form a picture of dead, grey tone, with some points of unfathomable blackness, and but few of redeeming colour—a life in which, surely, the last event would be looked forward to as the brightest.

Without at present sketching the biography further, we propose to examine the nature of the antecedents and events which influenced the formation of Miss Brontë's character; and then to review what that character was in itself, as represented by Mrs. Gaskell, and as set forth in "Jane Eyre;" and as we have given the first importance to hereditary influence, we shall first inquire what were the elements probably thence derived.

From her gentle, pious, Cornish mother, Miss Brontë inherited a refined, thoughtful, true womanly nature, and probably a delicate, fragile frame;—from her strong-willed, determined, Celtic father, other qualities which dominated over these.

Mr. Brontë was (or rather *is*, for the parent stem has survived all its branches) a native of Ireland, and came of a large family, remarkable for physical strength and beauty. He gave early tokens of great talent, combined with extraordinary force of will and general energy of character, as evinced by his separating himself from the hereditary agricultural pursuits, and devoting himself to an intellectual life. He opened a public school at sixteen years of age; afterwards became a private tutor; and, at the age of twenty-five, entered himself at Cambridge, and ultimately took holy orders.

He was a diligent pastor, but otherwise reserved in his intercourse with his people. He took an active and decided part in the machinery riots, in the early part of this century, in the manufacturing districts of Yorkshire; resisting popular clamour when unreasonable, but supporting the working men with all the energy of his nature when he thought them wronged. Mrs.

Gaskell describes him as of "strong, passionate Irish nature, generally compressed down with resolute stoicism—but it was there, notwithstanding all his philosophic calm and dignity of demeanour; though he did not speak when he was annoyed or displeased." The method which he adopted of working off superfluous excitement gives a little insight into some mental relations, which need not be more particularly specified when speaking of a living character. When annoyed seriously, he would fire pistols off in rapid succession out of the window, and so gradually calm down. He had other eccentricities of a still more suggestive nature, which must have had a marked effect upon the character of his family. He had imbibed the spirit of Rousseau and Day on education, and wished to make his children hardy, and indifferent to eating and dress. He allowed them no animal food during childhood; and on one occasion, when they had been walking over the moors, and their dry shoes and boots had been put before the fire ready for them, he burned them, lest they should become effeminate or vain. On another occasion he burned the hearth-rug, for reasons not given by the biographer; and cut a silk dress of Mrs. Brontë's in pieces, because he did not like it. He associated but little with his children, partly from choice, partly from other circumstances not necessary to detail; so that after their mother's death they were almost completely orphans. Mrs. Gaskell sums up his character as follows:—

"His opinions might be often both wild and erroneous, his principles of action eccentric and strange, his views of life partial and almost misanthropical; but not one opinion that he held could be stirred or modified by any worldly motive. He acted up to his principles of action; and if any touch of misanthropy mingled with his view of mankind in general, his conduct to the individuals who came in personal contact with him did not agree with such view. It is true that he had strong and vehement prejudices, and was obstinate in maintaining them, and that he was not dramatic enough in his perceptions to see how miserable others might be in a life that was all-sufficient to him; but I do not pretend to be able to harmonize points of character, and account for them, and bring them all into one consistent and intelligible whole. The family with whom I have now to do shot their roots down deeper than I can penetrate. I cannot measure them, much less is it for me to judge them. I have named these instances of eccentricity in the father, because I hold them to be necessary for a right understanding of the life of his daughter."

Referring to the well-known proverbial close alliances of great wit, we cannot doubt whence Charlotte Brontë's genius was derived. It is not difficult also to see, in the father's fiery, impetuous character, the source of the strong, indomitable will, the untiring energy, the deep, fervent imagination, and the reserve and isolation which characterized his daughter Charlotte.

Of Maria Branwell, afterwards Mrs. Brontë, but little is known. Mrs. Gaskell says that, "without having anything of her daughter's rare talents, Mrs. Brontë must have been, I should think, that unusual character, a well-balanced and consistent woman." Her surviving relatives speak of her as "meek and retiring, while possessing more than ordinary talents, which she inherited from her father; and her piety was genuine and unobtrusive." Those of her letters that remain, abound with tender womanly sentiment and grace. Her children seem to have inherited some portion of her admirable mental qualities, but all of that delicacy of constitution which removed her so early from their head.

This singularly assorted yet affectionate pair had six children, the very remarkable development of whose characters throws a strong reflected explanatory light upon that of the parents. They are described as grave and silent beyond their years; so quiet, that it would almost seem there were no children in the house. "Maria (only seven years old!) would shut herself up in the children's study with a newspaper, and be able to tell one everything when she came out," including debates in parliament, &c. &c. The four eldest sisters, in their very early life, produced the impression upon those who knew them, of "wild, strong hearts and powerful minds, hidden under an enforced propriety and regularity of demeanour and expression."

A dreary household and a dreary childhood must this have been. The mother was a constant invalid, and could take no active supervision of the children; not very anxious to see them either, "perhaps because the sight of them, knowing how soon they were to be left motherless, would have agitated her too much." Their father was much engaged, and "besides, he was not naturally fond of children, and felt their frequent appearance on the scene as a drag both on his wife's strength, and as an interruption to the comfort of the household." They had no young companions, and it does not appear that they had any "children's books;" but their "eager minds 'browsed undisturbed among the wholesome pasturage of English literature.'" And so from childhood they had their own society, their father's books, and the glorious wild moors which in after-days they loved so much, for their sole companions. Ah, those moors! When grim death visited the two eldest, it was only amid their wild scenery that they could hear his message; when far away from home, it was the wild sweep of the autumnal wind that sung in the ears of the wandering girls, and lulled them to sleep and dreams of their cheerless home, gilded into a fairy-palace of dreamland; it was only amid those moors that poor Emily Brontë could live—only on them that she could die; it was only there, where their first unchilled draughts of young life were drunk in, that they could again breathe it out, and yield it up to its great Author.

Maria Brontë, the eldest of these children, seems to have inherited a remarkable share of the best and finest qualities of both father and mother. Long before she died, at the age of eleven, "her father used to say that he could converse with her on any of the leading topics of the day, with as much freedom and pleasure as with any grown-up person." Mrs. Gaskell states that Helen Burns in "Jane Eyre" was a most faithful transcript of Maria's character, and that the conversations and incidents were such as actually did occur. We can scarcely imagine such sentiments as the following emanating from a mere child; but we quote them as indicating, if not her actual state of mind, yet the appreciation which Charlotte held of her sister's teaching and mode of thought at that time:—

"Life appears to me too short to be spent in nursing animosities or registering wrongs. We are and must be, one and all, burdened with faults in this world; but the time will come when, I trust, we shall put them off in putting off our corruptible bodies; when debasement and sin will fall from us with this cumbrous frame of flesh, and only the spark of the spirit remain,—the impalpable principle of life and thought, pure as when it left the Creator to inspire the creature: whence it came it will return,—perhaps again to be communicated to some being higher than man—perhaps to pass through gradations of glory, from the pale human soul to brighter, to the seraph! Surely it will never, on the contrary, be suffered to degenerate from man to fiend? No; I cannot believe that; I hold another creed, which no one ever taught me, and which I seldom mention, but in which I delight, and to which I cling, for it extends hope to all; it makes eternity a rest—a mighty home, not a terror and an abyss. Besides, with this creed, I can so clearly distinguish between the criminal and his crime; I can so sincerely forgive the first, whilst I abhor the last; with this creed revenge never worries my heart, degradation never too deeply disgusts me, injustice never crushes me too low: I live in calm, looking to the end."

We must give one further illustration of the characters of these children; for, as they were each others' sole associates, the mind of each is of importance as influencing all the rest. Mr. Brontë, apparently in one of his more social and domestic moods, wished to inquire into the talents and ideas of the children; and that they might, as he thought, speak more freely, he put a mask on the face of the one questioned. "I told them," says he, "to stand and speak boldly from under cover of the mask. I began with the youngest (Anne, afterwards Acton Bell, about fouryears old), and asked what a child like her most wanted; she answered, 'Age and experience.' I asked the next (Emily, afterwards Ellis Bell) what I had best do with her brother Branwell, who was sometimes a naughty boy; she answered, 'Reason with him; and when he won't listen to reason, whip him.' I asked Branwell what was the best way of knowing the difference between the intellects of man and woman; he answered, 'By considering the difference between them as to their bodies.' I then asked Charlotte what was the best book in the world; she answered, 'The Bible.' And what was the next best; she answered, 'The Book of Nature.' I then asked the next what was the best mode of education for a woman; she answered, 'That which would make her rule her house well.' Lastly, I asked the eldest (Maria, about ten) what was the best mode of spending time; she answered, 'By laying it out in preparation for a happy eternity.' I may not have given precisely their words, but I have nearly done so, as they made a deep and lasting impression on my memory. The substance, however, was exactly what I have stated."

Maria and Elizabeth Brontë died young,—aged eleven and ten,—and were buried at Haworth, near their well-beloved moors. Branwell lived to cast the shadow of a dark lurid cloud over almost the whole of his sister's life, by intemperance and all its concomitant vices. His wild, reckless, determined nature was shown to the very last—he insisted on dying standing up. Charlotte, Emily, and Anne were the Currer, Ellis, and Acton Bell that for a while formed the mystery of part of the literary world. Emily was of a strange, wild, fierce temper, permitting no sympathy, asking and accepting no aid. The portrait of "Shirley" is intended by Miss Brontë to represent what Emily might have been under favouring circumstances. The incident there related of the bite of the dog, and the cauterizing the wound with a red-hot iron, actually occurred to Emily. In her illness her sister thus writes of her: "Stronger than a man, simpler than a child, her nature stood alone. The awful point was, that, while full of ruth for others, on herself she had no pity; the spirit was inexorable to the flesh; from the trembling hand, the unnerved limbs, the fading eyes, the same service was exacted as they had rendered in health. To stand by and witness this, and not dare to remonstrate, was a pain no words can render." Dying of consumption, she would have no advice, no assistance, no sympathy. She refused to see a physician, or to take the remedies prescribed on her friends' descriptions. Only two hours before her death did she consent to see a medical attendant, and then it was too late.

Such a parentage—such family associations—are fully sufficient to account for the characteristic peculiarities of Charlotte Brontë; but there are still many other elements to notice, which combined to form this remarkable character. The impression made upon strangers by the aspect of the country and the house in which the Brontës resided is vividly shown in a letter from which the following is extracted :—

"The country got wilder and wilder as we approached Haworth; for the last four miles we were ascending a huge moor, at the very top of which lies the dreary, black-looking village of Haworth. The village street itself is one of the steepest hills I have ever seen, and the stones are so horribly jolting that I should have got out and walked if possible; but having once begun the ascent, to stop was out of the question. . . . . The clergyman's house was at the top of the churchyard,—so through that we went,—a dreary, dreary place, literally *paved* with rain-blackened tombstones, and all on the slope; for at Haworth there is on the highest height a higher still, and Mr. Brontë's house stands considerably above the church. There was the house before us, a small oblong stone house, with not a tree to screen it from the cutting wind; but how we were to get at it from the churchyard we could not see! There was an old man in the churchyard, brooding like a ghoul over the graves, with a sort of grim hilarity on his face. . . . . Presently we found ourselves in the little bare parlour. . . . . Joy can never have entered that house since it was first built; and yet, perhaps, when that old man married, and took home his bride, and children's voices and feet were heard about the house, even that desolate crowded graveyard and biting blast could not quench cheerfulness and hope. Now, there is something touching in the sight of that little creature entombed in such a place, and moving about, herself like a spirit, especially when you think that the slight still frame encloses a force of strong fiery life, which nothing has been able to freeze or extinguish."

A true wild, dreary moorland home was this Haworth, cut off from anything like easy communion with the surrounding district. A neighbour's visit was truly a journey; and so the village was almost isolated from the world, and the Brontës were almost isolated from the village. Here Miss Brontë, with comparatively short intervals of absence, passed the whole of her life; and little as was her intercourse with the people, it could not fail to have some influence upon her; and we shall now examine what this was likely to be.

"For a right understanding," says the biographer, "of the life of my dear friend Charlotte Brontë, it appears to me more necessary in her case than in most others that the reader should be made acquainted with the peculiar forms of population and society amidst which her earliest years were passed, and from which both her sisters' and her own first impressions of human life must have been received." Mrs. Gaskell describes the Yorkshiremen as endued with strong sagacity and a dogged power of will, which produce an air of self-sufficiency and independence "rather apt to repel a stranger." He rarely requires assistance, and rarely tenders it; hence he over-estimates his own powers.

"The practical qualities of a man are held in great respect; but the want of faith in strangers, and untried modes of action, extends itself even to the manner in which the virtues are regarded; and if they produce no immediate and tangible result, they are rather put aside as unfit for this busy, striving world, especially if they are more of a passive than an active character. The affections are strong, and their foundations lie deep; but they are not—such affections seldom are—wide-spreading, nor do they show themselves on the surface. Indeed there is little display of the amenities of life among this wild, rough population. Their accost is curt; their accent and tone of speech, blunt and harsh. Something of this may probably be attributed to the freedom of mountain air and of isolated hill-side life; something be derived from their rough Norse ancestry. They have a quick perception of character, and a keen sense of humour; their feelings are not easily roused, but their duration is lasting. Hence there is much close friendship and faithful service; and for a correct exemplification of the form in which the latter frequently appears, I need only refer the reader of 'Wuthering Heights' to the character of Joseph. These men are shrewd and faithful; persevering in following out a good purpose, fell in tracking an evil one. They are a powerful race both in mind and body, both for good and for evil."

Mrs. Gaskell gives many amusing and graphic illustrations of the rough energies and determined wills of these Haworth people, as well as occasionally of their brutalized nature, until we can see the origin of many of Miss Brontë's apparently most grotesque scenes and characters. We can see whence were derived the elements for the construction of such characters as the elder Crimsworth, Hunsdon, Yorke, Helstone, and Rochester; and also, from her limited intercourse with such men, how it was that her *dramatis personæ* became rather melodramatic than true natural men. For her imagination, when directed towards character and unchecked by experience, had a strong leaning to the melodrame. In her criticism this is strongly marked by her considering Walter Scott's "Varney" as one of the most powerful of his creations.

Many of the fierce riotous scenes described in "Shirley" are but graphic sketches of actual occurrences during Miss Brontë's childhood and youth; and we can testify that the savage brutality of the lower working classes is not at all overdrawn. We must add further, by way of completing the view of the moral medium amid which she was brought up, that ignorance and superstition, not to be exceeded by those of any heathen land, not only did prevail in some of these remote districts of Yorkshire during

Miss Brontë's youth, but do so still. We have ourselves met with children of advanced age who had never heard of a God, who had no idea what prayer meant, and were as thoroughly heathen as any New Zealander. In many parts, almost every door has a horse-shoe nailed to it, to keep out witches; and we have not unfrequently heard obstinate diseases attributed to the patient's having been "ill-wished" by some suspicious-looking old woman, who had undoubtedly been observed entering or leaving the house in some irregular manner, through keyhole or chimney. Notwithstanding all this, and much more that might be said on the same theme, Yorkshire is not *all* as represented by the Brontës: all the manufacturers are not Crimsworths—all the farms are not Wuthering Heights—all the gentlemen are not Rochesters—nor all the *plebs* Cherokees or Blackfoots. On the nature of the characters depicted by these three sisters Brontë, a recent writer in the "National Review" thus speaks:—

"How far the temperament, common under varied aspects to the three sisters, was due to the circumstances of their life, or how far to peculiarities of nature and race, it is impossible to determine. They seem curious offspring of the eccentric, strong-willed Irish father, and the simple, mild Cornish mother. It is as if the churchyard air they breathed, and the strong cold breezes from the moor, had entered into their very nature and made them what they were. Yet they were clearly not children of the soil; the glowing embers that lay but half smothered at the bottom of the character of at least two of them, had in it more of the Southern and Celtic element than of the Northman's opener, clearer fire. Half England now has formed an idea of Yorkshire on what these sisters have written: yet we doubt if they ever understood the North-country character. They studied its exceptional aspects, and familiarity with its external traits enabled them to give life-like costume to their pictures; but their narrow and secluded natures had neither the range nor the opportunity to grasp the broader characteristics of the people amongst whom they lived, and the North-country has received considerable injustice at their hands. . . . . The close shadow of the Brontës' churchyard home, the bitter winds, and the wild, dark aspect of their moors, have left the mark of their influence upon the writings as well as the characters of the sisters. They want softness, variety, beauty; they are too often dark, hopeless, discomfortable: on the other hand, they are vigorous and fresh, and bear welcome traces of Nature's close companionship with the minds from which they sprang. A personal impress is strongly marked on them. It is curious that though the writers all had strong imaginations, not one of them had the power to get rid for a moment of her own individuality. It permeates with its subtle presence every page they write. They were not engaging persons, and they felt that they were not—felt it acutely, and made others unduly sensible of it. Nor did they care to see others in their more agreeable and engaging aspects. They had been brought into close contact with the darker shades of character, and they studied them and reproduced them; too often they used light to give a greater depth to shadow, rather than shadow to set off light."

How Charlotte Brontë individually was affected by the varied influences to which we have alluded, will be best developed by a brief survey of her educational career, and her character during that time.

At a very early age she was sent to the establishment at Cowan Bridge, where events occurred which cast a deep gloom over her future life. Without entering into any of those details which have recently given origin to so much controversy, it is sufficient to know that the bad food and unhealthy situation of the place seemed to undermine the probably already delicate constitutions of the two elder sisters, Maria and Elizabeth: they did not die there, but were removed home, and died during the same year. And thus Charlotte, at the age of nine, was left the eldest of a motherless and almost fatherless family. She continued at the school a little longer, but was soon removed, as it appeared likely to injure her health. Whilst at school, she was considered a "bright, clever child;" but Mrs. Gaskell considers that the early part of the year 1825, just before the death of her sisters, was the last time in her life that the epithet "bright" could justly be applied to her. The active cares of life seemed to begin then; and with few intervals of relief, they continued thickening around her, enveloping her like a gloomy, impenetrable pall, till the time when she was left alone to her work, to wait, perhaps, with some impatience for the end. Now followed an interval of five years at home, in which Charlotte's tastes, tendencies, and character must have undergone great development. The children led a solitary, isolated life, in which there was much time for thought; they were thrown very much upon their own resources; their aunt, Miss Branwell, taught them what she could, and their father related to them public news which to him was interesting, and "from the opinions of his strong and independent mind they could gather much food for thought;" but all this could not exhaust the requirements of young, ardent, active minds. To this, in all probability, may be attributed the bias towards authorship which possessed their whole subsequent lives; for, even whilst such mere children, they adopted, almost from the necessity of their nature, the habit of inventing and writing tales, dramas, poems, &c., of which the principal subjects were the political and military celebrities of the day, especially the Duke of Wellington. These were sometimes combined into magazines, of which there was an editor, who received communications, criticisms, and letters upon the current topics of the time, and responded with due editorial gravity. Some of these productions are very remarkable for children. Another element, the results of which may be traced in all Miss Brontë's maturer writings, was furnished by Tabby, an elderly woman from the village, who came to reside with them as a sort of half servant, half *gouvernante*. Tabby was a relic of olden times—had lived in Haworth in the days of the packhorses and bells. "What is more, she had known the 'bottom,' or valley, in those primitive days when the fairies frequented the margin of the 'beck' on moonlight nights, and had known folk who had seen them. 'It wur the factories as had driven 'em away.' No doubt she had many a tale to tell of bygone days of the country-side : old ways of living, and former inhabitants ; decayed gentry who had melted away, and whose places knew them no more; family tragedies, and dark superstitious dooms; and, in telling these things without the least consciousness that there might be anything requiring to be softened down, would give at full length the bare and simple details." And so, at this plastic age, Charlotte's vivid imagination would become tinged unconsciously with that shade of superstition which every now and then would gleam forth amidst her strong, practical common sense ; and her mind would become stored with old legends and incidents, in which, in after-days, she would make her ideal characters actors. If we are not mistaken, even the grotesque incident connected with Mr. Rochester's insane wife was but a reproduction of a currently reported tale in that neighbourhood, with very slight modifications.

What a strange, irregular sort of culture these minds were undergoing, may be inferred, partly, from a list of painters which Charlotte, then only thirteen, made out, as those whose works she wished to see—including "Guido Reni, Julio Romano, Titian, Raphael, Michael Angelo, Correggio, Annibal Carracci, Leonardo da Vinci, Fra Bartolomeo, Carlo Cignani, Vandyke, Rubens, and Bartolomeo Ramerghi;" and this when she had probably never seen a painting worthy of the name.

When about fifteen, in 1831, she is described personally as "very small in figure, with soft, thick brown hair, and peculiar eyes, of which I find it very difficult to give a description, as she appeared to me in her later life." ("Jane Eyre" describes her own as green eyes.) "The usual expression was of quiet, listening intelligence; but now and then, on some just occasion for vivid interest or wholesome indignation, a light would shine out as if some spiritual lamp had been kindled which glowed behind those expressive orbs. . . . . As for the rest of her features, they were plain, large, and ill-set. . . . . Her hands and feet were the smallest I ever saw." . . . . "I can well imagine that the grave, serious composure which, when I knew her, gave her face the dignity of an old Venetian portrait, was no acquisition of later years, but dated from that early age when she found herself in the position of an elder sister to motherless children."

In January of this year she again went to school, where she remained nearly two years. She appeared at first as a timid, reserved, awkward, shortsighted girl—very deficient, indeed, in the ordinary branches of education, but confounding her companions, frequently and unconsciously, by her strange acquaintance with out-of-the-way matters that they knew nothing of. During this time, however, these defects of education were corrected, and some of the more amiable qualities of her mind evolved. When she left Roe Head, in 1832, she had "won the affectionate regard both of her teacher and her schoolfellows, and formed there the two fast friendships, which lasted her whole life long," with the "Mary" and the "E." of the memoir. Her correspondence with the latter forms a considerable portion of the work. Mrs. Gaskell observes:—

"In looking over the earlier portion of this correspondence, I am struck afresh by the absence of hope, which formed such a strong characteristic of Charlotte. . . . In after-life, I was painfully impressed with the fact that she never dared to allow herself to look forward with hope; that she had no confidence in the future; and I thought, when I heard of the sorrowful years she had passed through, that it had been this pressure of grief which had crushed all buoyancy of expectation out of her. But it appears from the letters, that it must have been, so to speak, constitutional; or perhaps the deep pang of losing her two elder sisters combined with a permanent state of bodily weakness in producing her hopelessness. If her trust in God had been less strong, she would have given way to unbounded anxiety at many a period of her life. As it was, we shall see, she made a great and successful effort to leave her 'times in His hands.'"

After remaining some time at home instructing her sisters, Charlotte returned to Roe Head as a teacher. Here her life was not unhappy until her health began to fail. At this time a sort of crisis occurred in her constitution, either physical or mental—for in such a tangled chain it is not possible to say which was the first link. "She told me" (says 'Mary') "that one evening she had sat in the dressing-room until it was quite dark, and then, observing it all at once, had taken sudden fright." A similar terror is alluded to in the earlier pages of "Jane Eyre."

"From that time her imaginations became gloomy or frightful; she could not help it, nor help thinking. She could not forget the gloom—could not sleep at night, nor attend in the day. She told me that one night, sitting alone, about this time, she heard a voice repeat these lines:—

'Come, thou high and holy feeling,
Shine o'er mountain, flit o'er wave,
Gleam like light o'er dome and sheeling'—

and eight or ten more lines which I forget. She insisted that she had not made them—that she had heard a voice repeat them. . . . Whether the lines were recollected or invented, the tale proves such habits of sedentary, monotonous solitude of thought, as would have shaken a feebler mind."

To the psychologist all these symptoms are familiar, and suggestive of a state of mind, the relations of which are not difficult to predicate. It is not at all improbable that Miss Brontë was subject occasionally to hallucinations of both eye and ear. Commenting, at a later period of her life, upon her habits of sitting up for hours after the family had retired, and when she was left alone by the death of her sisters, Mrs. Gaskell says:—

"No one on earth can even imagine what those hours were to her. All the grim superstitions of the North had been implanted in her during her childhood by the servants, who believed in them. They recurred to her now,—with no shrinking from the spirits of the dead, but with such an intense longing once more to stand face to face with the souls of her sisters, as no one but she could have felt. It seemed as if the very strength of her yearning should have compelled them to appear. On windy nights, cries, sobs, and wailings seemed to go round the house, as of the dearly-beloved striving to force their way to her. Some one conversing with her once objected, in my presence, to that part of 'Jane Eyre' in which she hears Rochester's voice crying out to her in a great crisis of her life, he being many, many miles distant at the time. I do not know what incident was in Miss Brontë's recollection when she replied, in a low voice, drawing in her breath, 'But it is a true thing; it really happened.'"

After this period, from time to time we hear of failing health, with the most fearful depression of spirits. "Mere bodily pain, however acute, she could always put aside; but too often ill-health assailed her in a part far more to be dreaded. Her depression of spirits, when she was not well, was pitiful in its extremity. She was aware that it was constitutional, and could reason about it; but no reasoning prevented her suffering mental agony, while the bodily cause remained in force."

In 1850 she herself says:—

"I don't know what heaviness of spirit has beset me of late, made my faculties dull, made rest weariness, and occupation burdensome. Now and then the silence of the house, the solitude of the room, has pressed on me with a weight I found it difficult to bear, and recollection has not failed to be as alert, poignant, and obtrusive as other feelings were languid."

Some time afterwards, there is a long annual period of this sort of affliction. To a friend she writes:—

"Sometimes the strain falls on the mental, sometimes on the physical part of me; I am ill with neuralgic headache, or I am ground down to the dust with deep dejection of spirits. That weary time has, I think and trust, got over for this year. It was the anniversary of my poor brother's death, and of my sisters' failing health: I need say no more."

Yet amid all this she was working on, sometimes attempting the life of a private governess, sometimes in a school, with the physical and mental results that might be anticipated. But circumstances seemed to make it imperative that the girls should do something to relieve their father from their support, particularly as at this time he was threatened with blindness. A scheme was devised for opening a school, as a preparation for which, Charlotte and Emily entered for a while a *pensionnat* at Brussels. Of their life at this time, a sufficiently accurate idea may be gathered from "Villette" and "The Professor." The scenes and mode of tuition described in these works seem to be almost historical—all save the actual personal incidents. It was here that, under the philosophic tuition and guidance of M. Heger, Miss Brontë's powerful thinking faculties were, for the first time, *systematically* drawn out and cultivated. It is not impossible that here also, for the first time, she learnt the potency of other and yet untried emotional elements of her nature; but of this we know nothing, save by perhaps falsely interpreted inferences—so that nothing can be founded on the supposition. On her final return home from Brussels, in January, 1844, it appears that the project of a school was given up temporarily, on account of Mr. Brontë's increasing blindness, and his daughter's conviction that she must not leave him. Shortly after this, she writes to a friend:—"I do not know whether you feel as I do, but there are times now when it appears to me as if all my ideas and feelings, except a few friendships and affections, are changed from what they used to be; something in me which used to be enthusiasm, is tamed down and broken. I have fewer illusions: what I wish for now is active exertion—a stake in life. Haworth seems such a lonely, quiet spot, buried away from the world. I no longer regard myself as young—indeed, I shall soon be twenty-eight; and it seems to me as if I ought to be working and braving the rough realities of the world, as other people do. It is, however, my duty to restrain this feeling at present, and I will endeavour to do so." Poor soul! her discipline was harder than any work—a weary waiting and watching for opportunities that came not—standing well-nigh alone, the earth as iron and the heavens as brass—youth passed without happiness—age creeping on without hope—a weary lot indeed.

In 1845, a volume of poems were published by "Currer, Ellis, and Acton Bell." They attracted but little attention—that little not very favourable: another disappointment. Charlotte quietly submits to it:—"The book was printed: it is scarcely known; and all of it that merits to be known are the poems of Ellis Bell. The fixed conviction I held, and hold, of the worth of these poems, has not indeed received the confirmation of much favourable criticism; but I must retain it notwithstanding." Then each of the sisters wrote a tale—"The Professor," "Wuthering Heights," and "Agnes Grey." After many unsuccessful attempts, the two latter found a publisher; but Charlotte's tale was always rejected. "Currer Bell's book," she writes, "found acceptance nowhere, nor any acknowledgment of merit, so that something like the chill of despair began to invade his heart." But, undaunted by this, the brave woman began "Jane Eyre," the work which has placed her in the first rank of female novelists. It was published on October 16th, 1847. And now her fame was made—the worst struggle was over; but clouds gather thick and fast around the domestic life of the author, one by one in rapid succession, the wild, reckless brother, the dearly-loved sister Emily, and the youngest and gentlest—the most like the mother—Anne, passed away, leaving the subdued, broken-spirited Charlotte to labour on alone, and await her own time, uncheered by her constant companions in weal or woe. By the 28th of May, 1849, she is alone:—

"My life (she writes) is what I expected it to be. Sometimes when I wake in the morning, and know that Solitude, Remembrance, and Longing are to be almost my sole companions all day through—that at night I shall go to bed with them—that they will long keep me sleepless—that next morning I shall wake to them again—sometimes I have a heavy heart of it. But crushed I am not, yet; nor robbed of elasticity, nor of hope, nor quite of endeavour. I have some strength to fight the battle of life. I am aware, and can acknowledge, I have many comforts, many mercies. Still I can *get on*. But I do hope and pray that never may you, or any one I love, be placed as I am. To sit in a lonely room—the clock ticking loud through a still house—and have open before the mind's eye the record of the last year, with its shocks, sufferings, and losses, *is* a trial."

Mrs. Gaskell gives many features in detail, but no complete portrait of the character formed by this life of patient, unbroken endurance. If we examine her correspondence and writings generally, we find unmistakeable marks of an ardent, fiery temperament, constantly repressed—of an imagination vivid, scintillating, wild, and *eerie*, working upon limited material and experience—of a disposition warm and affectionate, abounding with all manner of human sympathy, that could never find a vent or fit sphere for expansion—of a piety deep, sincere, and practical, yet eminently undemonstrative and retiring, shunning all question or observation—of a habit of mind reserved, timid, and observant—of an entire noble nature, enthusiastic and aspiring, quelled and worn down with unremitting care and suffering. But for the possible expansions of such a nature, we must look further than her confined, isolated, suffering life. We must see her as she represents herself acting and feeling in imaginary conjunctures; and we need not fear taking her own testimony, for she was not one to flatter even self, and under the guise of a fictitious name. For this purpose we must glance over the prominent points of "Jane Eyre," in which she appears to have given a daguerreotype of herself;* the veritable thoughts and feelings of her life being hung on imaginary incidents. Jane is first known as a little plain, shy orphan girl of ten years old, neglected and ill-treated by an aunt, "bullied and punished" by her son, scorned and repelled by her daughters; constantly looking for something or some one to love, but in vain; utterly unattractive in appearance and manners. At last, in response to more than usually cruel treatment, the ardent impetuous nature flames out, and overwhelms the hard ruthless aunt with fear and astonishment. She is sent to school at the establishment of Lowood (Cowan Bridge), for clergymen's daughters, where for eight years she remains utterly cast off by her relatives—six years as a pupil, and two as a teacher; indicating, as is observed afterwards, a great tenacity of life, considering the treatment which is there described. Unjustly accused of faults not her own, she is at first morose and rebellious in temper; but the pressure of constant discipline, and some little casual kindness, at last reduce her to an ordinary tolerably-conducted school-girl. At the end of eight years this isolated life becomes unendurable, and she advertises for a situation as governess, determined to see more of her fellow-creatures than a girls'-school can exhibit. Mrs. Fairfax, the housekeeper of a country gentleman, Mr. Rochester, answers her advertisement, and engages her to take charge of a ward of her master's. The first meeting between Jane and Mr. Rochester is arranged in a dark lane, where his horse falls—an accident which he afterwards lays to the charge of "her people," as he calls the fairies. Now this Mr. Rochester is evidently a pet invention of Miss Brontë's, and is intended to represent a fine, noble, generous English country gentleman, with a decided leaning towards unconventionalities. In nothing scarcely is Miss Brontë's contracted intercourse with society more manifest than in her elaborate delineations of character; her sketches are good,

* At the time of the first appearance of "Jane Eyre," during its first burst of popularity, a relative of the writer was asked by a friend if she had ever seen any one like Jane; to which she replied, that many years before she had seen one, and only one person who accurately resembled the character; that was a Miss Brontë. She had seen her but once, but the resemblance was too vivid to be mistaken even at that distance of time.

but her finished portraits are chiefly caricatures. This same Mr. Rochester is, we are sorry to say, nothing more nor less than a selfish, coarse, not far from brutal sensualist—a melodramatic hero, with some of the reflected refinement of the beholder upon him. He has on the third story of Thornfield a mad wife, described as several degrees worse than a satyr, and more savage than a hyena; but of this no one knows anything, and he would willingly have had Jane for his second wife. In their first social interview, he inquires with a domineering abruptness into her previous life and qualifications, which he criticises with no sparing hand; but Jane always likes to be "bullied." He examines her water-colour drawings, which meet with more favour than her other performances. Three of these he selects to analyse; and they give so vivid a view of Miss Brontë's style of imagination, that we quote the description :—

"The first represented clouds low and livid, rolling over a swollen sea; all the distance was in eclipse; so, too, was the foreground, or rather the nearest billows, for there was no land. One gleam of light lifted into relief a half-submerged mast, on which sat a cormorant, dark and large, with wings flecked with foam; its beak held a gold bracelet, set with gems. Sinking below the bird and mast, a drowned corpse glanced through the green water; a fair arm was the only limb clearly visible, whence the bracelet had been washed or torn.

"The second picture contained for foreground only the dim peak of a hill, with grass and some leaves slanting as if by a breeze. Beyond and above spread an expanse of sky, dark blue, as at twilight; rising into the sky was a woman's shape to the bust, portrayed in tints as dusk as I could combine. The dim forehead was crowned with a star; the lineaments below were seen as through the suffusion of vapour; the eyes shone dark and wild; the hair streamed shadowy, like a beamless cloud torn by storm or by electric travail. On the neck lay a pale reflection like moonlight; the same faint lustre touched the train of thin clouds from which rose and bowed this vision of the Evening Star.

"The third showed the pinnacle of an iceberg piercing a polar winter sky; a muster of northern lights reared their dim lances, close serried along the horizon. Throwing these into distance, rose, in the foreground, a head—a colossal head, inclined towards the iceberg, and resting against it. Two thin hands, joined under the forehead, and supporting it, drew up before the lower features a sable veil; a brow quite bloodless, white as bone, and an eye hollow and fixed, blank of meaning but for the glassiness of despair, alone were visible. Above the temples, amidst wreathed turban folds of black drapery, vague in its character and consistency as cloud, gleamed a ring of white flame, gemmed with sparkles of a more lurid tinge. This pale crescent was 'The likeness of a Kingly Crown;' what it diademed was 'the shape which shape had none.'"

Nothing ever impressed us more strongly than this with the conception of Miss Brontë's thoughtful and lonely habits of mind, nor of her wonderful power of word-painting. In the next interview of Jane with her master, as she loves to call him, they come to personalities:—

"'You examine me, Miss Eyre: do you think me handsome?'

"The answer somehow slipped from my tongue before I was aware, 'No, sir.'

"'Criticize me: does my forehead not please you?'

"He lifted up the sable waves of hair which lay horizontally over his brow, and showed a solid enough mass of intellectual organs, but an abrupt deficiency where the suave sign of benevolence should have risen. 'Now, ma'am, am I a fool?'

"'Far from it, sir. You would, perhaps, think me rude if I inquired in return whether you are a philanthropist?'"

The conversations between these two singular characters are piquant enough. Jane's answers, however, remind us much more strongly of what occurs to a naturally shy person afterwards as what might have been said, than of the sort of repartee that was likely actually to pass between two such interlocutors.

The story we cannot follow step by step. Jane loves Mr. Rochester, and he loves her with frantic passion. Mr. Rochester is Jane without her principle, and supplemented by a strong, coarse, vigorous nature, moral and physical—hence their coherence. He relates early in their acquaintance, with wonderful candour, the story of his early sensualities, to which she listens with equanimity, and ultimately undertakes the task of his guardian angel, by promising to marry him. Before all the misfortunes of her life, she has been warned by dreams of carrying about a helpless child; and for some time before the wedding-day this vision haunts her. On the morning of the day, in the church itself, the secret of the former (or present) mad wife is revealed, and the ceremony, of course, is stopped.

The succeeding scenes are painted with a power which few writers can equal, scarce any excel; but Jane's unbending principle triumphs over all the passion and sophistry of her lover, and she escapes. For days she undergoes the most sickening privations, but finds a home and friends at last, with whom she lives a year. At a crisis of her history she hears Mr. Rochester's voice calling, "Jane, Jane, Jane," though he is a hundred miles distant: she answers, "I am coming—wait for me;" and it appears ultimately that he also heard the answer. The next day she sets off for Thornfield, which she finds burnt down. The wife was burnt with it, and Mr. Rochester was maimed and blinded at the time. He is absent, but she follows him, and they are married; his sight partly returns, and for ten years their happiness is perfect.

"I have now been married ten years. I know what it is to live entirely for and with what I love best on earth. I hold myself supremely blest beyond what language can express; because I am my husband's life as fully as he is mine. No woman was ever nearer to her mate than I am; never more absolutely bone of his bone, and flesh of his flesh. I know no weariness of my Edward's society; he knows none of mine; any more than we each do of the pulsation of the heart that beats in our separate bosoms—consequently we are ever together. To be together is for us to be at once as free as in solitude, as gay as in company. All my confidence is bestowed on him, all his confidence is devoted to me; we are precisely suited in character—perfect concord is the result."

Alas! that one who so well could paint what life might be, should have so little experience of it. When her youth was passed, Miss Brontë married;—whether with that perfect, expansive, all-comprehensive love of which her original nature was capable or not, we have no right to inquire. She was happy. "We her loving friends, standing outside, caught occasional glimpses of brightness, and pleasant, peaceful murmurs of sound, telling of the gladness within; and we looked at each other, and said, 'After a hard and long struggle—after many cares and many bitter sorrows—she is tasting happiness now!' Those who saw her, saw an outward change in her look, telling of inward things. And we thought, and we hoped, and we prophesied in our great love and reverence. . . . But God's ways are not as our ways!"

In a few short months she went to join those sisters whom she had loved so well. On March 31st, 1855, she rested at last, after a weary warfare of thirty-nine years. *Requiescat.*

---

